# The impact of resilience on psychological outcomes in women with threatened premature labor and spouses: a cross-sectional study in Southwest China

**DOI:** 10.1186/s12955-017-0603-2

**Published:** 2017-01-31

**Authors:** Chunhua Nie, Qin Dai, Ren Zhao, Yushu Dong, Yushan Chen, Hui Ren

**Affiliations:** 1 School of Nursing, Third Military Medical University, No.30 Gaotanyan Street, Chongqing, 400038 China; 2 Psychological Nursing Office, School of Nursing, Third Military Medical University, No.30 Gaotanyan Street, Chongqing, 400038 China; 3 Department of Cardiology, General Hospital of Shenyang Military Region, No.83 Wenhua Road, Shenyang, 110016 China; 4 Department of Neurosurgery, General Hospital of Shenyang Military Region, No.83 Wenhua Road, Shenyang, 110016 China

**Keywords:** Threatened premature labor, Family resilience, Depression, Psychological outcomes

## Abstract

**Background:**

Threatened premature labor (TPL) is a severe obstetric complication which affects the mental and physical health of both the mother and fetus. Family resilience may have protective role against psychological distress in women experiencing these pregnancy complications. There may be resilience related risk factors in TPL women, and interplays may exist among psychological variables and within couples. This study aims to examine psychological outcomes influenced by different levels of resilience, and explore psychological interactions in TPL women, spouses, and between women and spouses.

**Methods:**

Six validated questionnaires were used to measure the psychological outcomes (Connor-Davidson resilience scale CD-RISC, Edinburgh postnatal depression scale EPDS, positive and negative affect scale PANAS, pregnancy pressure scale PPS, simplified coping style questionnaire SCSQ, social support rating scale SSRS) in 126 TPL women hospitalized in three tertiary hospitals and 104 spouses in Southwest China.

**Results:**

Low resilient women had significantly more complicated placenta praevia, longer pediatric observation, more pressure than high resilient women. They also had significantly less active coping and positive affect, more negative affect and depression compared to high resilient women and their spouses. Although the socio-demographic characteristics of both TPL women and spouses and psychometric parameters of spouses had no significant differences, the prevalence rates of depression in spouses were notable. Compared with spouses, TPL women had a more complex interaction among these psychometric factors, with women’s resilience negatively associated with their partners’ negative affect, and their pressure positively correlated with pressure and negative affect of spouses.

**Conclusions:**

Pregnancy complicated with placenta praevia and pediatric observation may be risk factors for resilience of women with TPL. Maternal resilience has an important impact on the psychological outcomes in TPL women. A screening for resilience, depression and other psychological outcomes in couples with TPL and early psychological intervention of low resilient couples may be appropriate to promote resilience and well-being of these families.

**Electronic supplementary material:**

The online version of this article (doi:10.1186/s12955-017-0603-2) contains supplementary material, which is available to authorized users.

## Background

Threatened premature labor (TPL) is a high-risk complication in pregnancy that not only has detrimental impact to the health of pregnant women, but could also lead to neonatal death, cerebral palsy, cognitive impairment, blindness, deafness, respiratory illness, and neonatal care complications [1–6]. Thus, TPL poses a significant public health issue, with implications for child and family well-being, including impact on the psychological well-being of expectant mothers and fathers [5, 7–9].

Family resilience refers to the characteristics, dimensions and properties of families, which help families to be resilient to disruption in the face of change and be adaptive in the face of crisis situations [10]. For all families, pregnancy is a period which may potentially create additional stressors. Pregnancies complicated with TPL pose chronic stressors due to the specific pathophysiological course of TPL, thus exhausting already limited resources available to these families. In an era of scarce resources, intervening to strengthen family resilience is of particular interest as it enables families to care for their own members. In addition, for women of childbearing age, the concept of family resilience may be particularly salient as the woman’s partner likely represents her closest form of intimate support. However, the resilience of families with TPL, including that of expectant father is currently unclear. Understanding the factors associated with family resilience may provide important insight into effectively support childbearing families experiencing TPL.

Although maternal mental health problems have been extensively studied and addressed to be a significant health problem, the majority of these studies have been focused on postpartum women [11–15], with very limited research on the antenatal period [16]. On the other hand, although it is widely recognized that paternal mental illness could increase the risk of behavioral and emotional problems in children [17, 18], paternal mental status during pregnancy is largely under-researched. A few pioneering longitudinal studies regarding expectant fathers’ depression [19–21] stated that expectant father demonstrated more symptoms of distress, including becoming more depressed and irritable as well as having more negative affect in the postnatal period. However, the scenario regarding paternal depression and other mental problems during the antenatal period, especially in high risk pregnancies such as TPL remains largely unknown. Knowledge on the possible psychological interactions between pregnant women and their spouses is also scarce.

We hypothesized the existence of risk factors for the resilience of TPL women, and possible psychological associations might exist among resilience and other psychometric factors and within couples. The present study aims to identify possible risk factors that contribute to the level of resilience of TPL women, and investigate interactions of psychological factors in TPL women, spouses, and between women and spouses.

## Methods

### Participants and study design

This study was conducted in the inpatient unit for the prevention of TPL in three tertiary hospitals/Medical Centers in Chongqing of Southwest China. TPL women at 28 to 37 weeks of gestation (*n* = 126) and the majority of their spouses (*n* = 104) were invited to participate in the study. Three hospitals/Medical Centers were Xinqiao Hospital and Daping Hospital affiliated with the Third Military Medical University (TMMU), and Chongqing Obstetrics and Gynecology Hospital/Institute for Genetic and Reproductive Medicine in Chongqing. Inclusion criteria were pregnant women hospitalized with the diagnosis of threatened premature labor (ICD-9-CM 644.03), and fetuses were alive without detected deformity or defect by ultrasound. Women or spouses with previous diagnosed psychiatric disorders were excluded.

Eligible participants were approached from March 1^st^ to August 1^st^ 2016, all TPL women were followed up at 6–8 weeks postpartum in this study, and information regarding duration of pediatric observation was collected. After explaining the purpose of the study, participants received an informed consent form and the Chinese questionnaire battery. No financial compensation was offered to the women or spouses for their participation.

The study protocol was approved by the Ethics Committee of TMMU, and informed, written consents were obtained from all participants.

### Measures

Six validated questionnaires were used to measure resilience, pregnancy pressure, coping style, social support, depression and affect. Social-demographic and reproductive characteristics were collected, including age, height, weight, smoking and drinking history, residence, education, monthly income (in Chinese Currency, Yuan), occupation categories, type of pregnancy, way of conception, gravidity, pregnant complications, fetus protection, and neonatal outcomes.

The 25 items of the **Connor Davidson Resilience Scale (CD-RISC)** are each scored on a five-point scale, with high scores indicating greater resilience levels [22]. The CD-RISC has sound psychometric properties, good internal consistency (Cronbach’s alpha 0.89) and test retest reliability (intra-class correlation coefficient of 0.87) [22], and was also used in Chinese population [23, 24]. The 25 items are subdivided into three factors, including hardiness (13 items), strength (8 items), and optimism (4 items). For this study, women with low resilience were defined as having a score of < 50. This cutoff point was based on score quartiles, with the lowest 25% of the scores defined as low resilience [25].

The **Pregnancy Pressure Scale (PPS)** is a 30-item scale measuring the source and extent of pregnancy related pressure of both the pregnant woman (alpha = 0.84) and spouse (alpha = 0.85) [26]. Participants respond to the items regarding how much they agree with the different statements from 0 “no pressure” to 3 “severe pressure”. All items were added to a total pressure score, with higher scores indicting higher levels of pressure. The statements are categorized into four causal factors of pressure, including parenthood recognition (15 items), assurance of the health and safety of mother and fetus (8 items), changes of body shape and physical activities of mother (4 items),and others (3 items). 3 items assigned as others include “concern the ability to rear child properly”, “concern spouses mutual affection after having a baby”, and “concern unable to give the child a good support”. The PPS has demonstrated an acceptable reliability among Chinese pregnant women and spouses [26, 27].

Coping style was measured by a **Simplified Coping Style Questionnaire (SCSQ)**. This questionnaire was developed by Xie YN [28] based on the Ways of Coping questionnaire by Folkman and Lazarus [29]. It is a 20-item self-report questionnaire that includes two dimensions, active coping (12 items) and passive coping (8 items), with higher scores representing greater active/passive coping manners. Participants were asked to agree or disagree on a 4-point Likert scale according to how frequently they adopt on each item from 0 “never” to 3 “very often”. The instrument has been commonly used in Chinese and the Cronbach’s alpha coefficients for the two dimensions were 0.80 and 0.73, respectively [30].

The Chinese version of the **Social Support Rating Scale (SSRS)** is comprised of 10 items and was originally developed in China by Xiao [31]. This scale was used to determine the objective support (3 items), subjective support (4 items), and availability (3 items) of social support from family, friends and significant others, with higher scores indicating better social support. The SSRS has been used in a wide range of Chinese populations due to its high reliability and validity [32–35], with two month test-retest reliability to be 0.92.

The **Edinburgh Postnatal Depression Scale (EPDS)** [36] was used to assess depressive symptoms in the postnatal periods. It is a self-report measure consisting of 10 items and each item is rated on a 4-point scale. It is a well-validated and the most widely used screening measure of postpartum depression among women. It has also been validated for use in the antenatal period [37] and among men as measurement of paternal depression [38]. The Chinese version of the EPDS has been validated among pregnant women with satisfactory psychometric properties [39]. The recommended cut-off of 13 was used to define a probable case of depression [17]. Cronbach’s alpha for the EPDS is 0.87 [36].

The 20-item **Positive and Negative Affect Scale (PANAS)** [40] measures affects of participants during the past 1–2 weeks. Respondents were asked how much they agree on the statements of affects, with responses from 1 “not at all” to 5 “extremely”. The items are summarized into positive affect (10 items, alpha = 0.85) and negative affect (10 items, alpha = 0.83), with higher scores representing more positive/negative affect. The PANAS has been validated in Chinese populations [41, 42].

### Statistical analyses

Data were analyzed using GraphPad Prism version 6.0 and the statistical package for the social sciences (SPSS) version 17.0 softwares. The independent samples *t*-test was conducted to test for differences in age, height, weight, duration of fetus protection, gestational days, duration of pediatric observation, and all the psychometric scores. The chi-square test was used to test for differences in the status of smoking, drinking, residence, education, monthly income, occupation categories, type of pregnancy, conception, gravidity, complications, previous history of fetus protection, pediatric observation and depression status. Among these samples, if the frequencies were less than 5, Fisher’s exact test was calculated. The Spearman’s Rho estimated correlations between psychological factors. The level of significance was determined with *p* < 0.05.

## Results

There were no significant differences in socio-demographic characteristics between low and high resilient TPL women, as well as between spouses divided by resilient level of women, including age, height, smoking and drinking history, residence, educational levels, monthly income, and occupational categories (Table 1). Comparison of socio-demographic data between spouses and TPL women showed high resilient TPL women were significantly younger, had less body weight, and had lower proportion of work in enterprise or in the management post than their partners (Additional file 1: Table S1). The income level of high resilient TPL women had higher proportion in 1000–1999 Yuan and lower proportion in 2000 to 2999 Yuan per month than their spouses (Additional file 1: Table S1).

**Table 1:** Socio-demographic characteristics within women with TPL by level of resilience and within spouses

	Low silient women (*n* = 32) *n* (%)	High resilient women (*n* = 94) *n* (%)	*p*	Spouses of low resilient women (*n* = 27) *n* (%)	Spouses of high resilient women (*n* = 77) *n* (%)	*p*
Age (years)	30.7 ± 0.9	29.4 ± 0.4	0.16	31.4 ± 0.9	31.2 ± 0.8	0.87
Height (cm)	159.1 ± 0.8	158.2 ± 1.2	0.65	167.9 ± 1.3	167.4 ± 1.6	0.84
Weight (kg)	63.8 ± 1.6	65.2 ± 0.9	0.46	67.7 ± 2.1	69.3 ± 1.8	0.65
Smoker						
Yes	0 (0.0)	6 (6.4)	0.34	14 (51.9)	42 (54.5)	0.81
No	32 (100)	88 (93.6)		13 (48.1)	35 (45.5)	
Drinker						
Yes	0 (0.0)	5 (5.3)	0.33	12 (44.4)	38 (49.4)	0.66
No	32 (100)	89 (94.7)		15 (55.6)	39 (50.6)	
Residence						
Urban	21 (65.6)	67 (71.3)	0.55	19 (70.4)	50 (64.9)	0.78
Rural	11 (34.4)	27 (28.7)		8 (29.6)	27 (35.1)	
Education						
Middle school	5 (15.6)	16 (17.0)	0.85	4 (14.8)	12 (15.5)	1.00
High school/TSS	6 (18.7)	25 (26.6)	0.37	5 (18.6)	26 (33.8)	0.14
Junior college	15 (46.9)	35 (37.2)	0.34	9 (33.3)	19 (24.7)	0.38
University	6 (18.8)	18 (19.2)	0.96	9 (33.3)	20 (26.0)	0.46
Monthly income						
< 1000	3 (9.4)	8 (8.5)	0.69	0 (0.0)	2 (2.6)	1.00
1000-1999	7 (21.9)	18 (19.1)	0.56	3 (11.1)	5 (6.5)	0.42
2000-2999	8 (25.0)	18 (19.1)	0.33	7 (25.9)	25 (32.4)	0.53
3000-4999	10 (31.2)	27 (28.7)	0.79	9 (33.3)	20 (26.0)	0.46
5000-9999	0 (0.0)	8 (8.5)	0.20	5 (18.6)	15 (19.5)	0.91
Others	4 (12.5)	15 (16.1)	0.78	3 (11.1)	10 (13.0)	1.00
Occupation categories						
Government/Military	2 (6.2)	5 (5.3)	1.00	1 (3.7)	2 (2.6)	1.00
Enterprise/Management	2 (6.2)	4 (4.3)	0.64	6 (22.2)	12 (15.6)	0.43
Office	6 (18.8)	24 (25.5)	0.44	8 (29.6)	15 (19.5)	0.27
Education/Science	4 (12.5)	8 (8.5)	0.50	3 (11.1)	6 (7.8)	0.69
Healthcare	0 (0.0)	1 (1.1)	1.00	0 (0.0)	3 (3.9)	0.57
Industry/Service	3 (9.4)	18 (19.2)	0.28	3 (11.1)	11 (14.3)	1.00
Private business	2 (6.2)	9 (9.6)	0.73	1 (3.7)	12 (15.5)	0.18
Others	13 (40.7)	25 (26.6)	0.14	5 (18.6)	16 (20.8)	0.80

*TPL*, threatened premature labor; *TSS*, technical secondary school

As to the reproductive characteristics, Table 2 shows that low resilient women had higher proportion of placenta praevia, and longer pediatric observation time recorded 6–8 weeks postpartum than high resilient women.

**Table 2:** Reproductive characteristics of low and high resilient women with TPL

	Low resilience (*n* = 32) *n* (%)	High resilience (*n* = 94) *n* (%)	*p*
Planned pregnancy			
Yes	26 (81.3)	74 (78.7)	0.76
No	6 (18.7)	20 (21.3)	
Conception			
Natural	29 (90.6)	89 (94.7)	0.42
via IUI/IVF	3 (9.4)	5 (5.3)	
Gravidity			
Once	14 (43.8)	50 (53.2)	0.36
More than once	18 (56.2)	44 (46.8)	
Complications			
PROM	6 (18.8)	28 (29.8)	0.22
GDM	4 (12.5)	11 (11.7)	1.00
ICP	2 (6.3)	17 (18.1)	0.15
Gestational hypertension	2 (6.3)	7 (7.4)	1.00
Placenta praevia	8 (25.0)	10 (10.6)	<0.05
Twin pregnancy	5 (15.6)	9 (9.6)	0.35
Others	5 (15.6)	15 (16.0)	0.96
Fetus protection			
Duration (days)	7.5 ± 1.2	7.1 ± 0.8	0.77
Gestational days	228 ± 3	229 ± 2	0.72
Previous history			
Yes	8 (75)	22 (76.6)	0.85
No	24 (25)	72 (23.4)	
Pediatric observation			
Yes	12 (37.5)	26 (27.7)	0.29
No	20 (62.5)	68 (72.3)	
Duration (days)	15.7 ± 2.3	10.4 ± 1.0	<0.05

*GDM*, gestational diabetes mellitus; *ICP*, intrahepatic cholestasis of pregnancy; *IUI*, intrauterine insemination; *IVF*, in-vitro fertilization; *PROM*, premature rupture of membranes; *TPL*, threatened premature labor

**Fig. 1:**
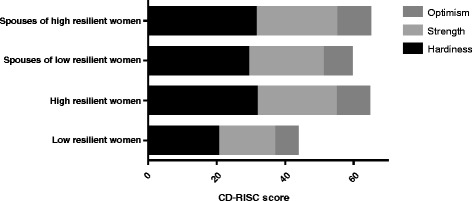
Comparison of resilience in TPL women and spouses. Low resilient women had significantly lower scores in optimism, strength, and hardiness than high resilient women and spouses (*P* < 0.0001 for all comparisons). Spouses in both groups had similar scores in all three components

The psychological measures in Fig. 1 reveal that low resilient women had significantly lower scores in all three components of resilience, including hardiness, strength, and optimism than their spouses and high resilient TPL women. Compared to high resilient women, they also reported to have higher pressures in pregnancy, triggered by parenthood recognition, concerns of the health and safety of themselves and the fetus. Higher socres were also found in other factors of low resilient women, including whether they can rear the child properly, whether pregnancy would affect mutual affection of husbands and wives, and uncertainty about child support in the future. Low resilient TPL women also had a lower mean score of active coping than high resilient women. Accordingly, depression symptoms were more severe in women with TPL of low resilience, with more depressed women found in low resilient group than high resilient group (50.0% *vs* 27.7%, *p* < 0.05) based on a cutoff value of EPDS score ≥ 13. In addition, less positive affect and more negative affect were found in low resilient women compared to high resilient TPL women (Table 3). Comparison of psychometric characteristics between spouses shows no significant difference in every measure and its components, including resilience, pressure, coping style, social support, depression, and affect (Fig. 1 & Table 3).

**Table 3:** Psychometric factors of women with TPL divided by low and high resilience and of spouses

	Low resilient women (*n* = 32) *n* (%)	High resilient women (*n* = 94) *n* (%)	*p*	Spouses of low (*n* = 27) *n* (%)	Spouses of high resilient women resilient women (*n* = 77) *n* (%)	*p*
Pregnancy pressure (PPS)	65.4 ± 2.5	56.1 ± 1.5	<0.01	60.3 ± 3.0	54.3 ± 1.6	0.06
Parenthood recognition	26.6 ± 1.1	22.1 ± 0.7	<0.01	23.2 ± 1.3	21.1 ± 0.7	0.14
Health/safety of mother/fetus	24.8 ± 1.0	21.0 ± 0.7	<0.01	22.0 ± 1.4	20.1 ± 0.8	0.23
Body shape/activity change	9.6 ± 0.7	8.3 ± 0.4	0.11	9.1 ± 0.9	8.5 ± 0.7	0.68
Other factors						
Rear child properly	2.6 ± 0.2	2.0 ± 0.1	<0.01	2.2 ± 0.2	2.3 ± 0.2	0.91
Spouses mutual affection	1.7 ± 0.1	1.4 ± 0.1	<0.05	1.4 ± 0.1	1.4 ± 0.1	0.92
Child support	2.7 ± 0.2	1.9 ± 0.1	<0.0001	2.1 ± 0.2	1.9 ± 0.1	0.39
Coping style (SCSQ)						
Active coping	19.1 ± 0.9	22.5 ± 0.6	<0.01	22.3 ± 1.1	21.8 ± 0.9	0.77
Passive coping	11.1 ± 0.8	11.3 ± 0.5	0.84	12.4 ± 1.1	11.9 ± 0.7	0.66
Social support (SSRS)	38.4 ± 1.2	41.3 ± 0.8	0.06	40.2 ± 1.6	38.5 ± 1.0	0.35
Objective support	9.9 ± 0.9	10.7 ± 0.4	0.35	11.8 ± 1.2	10.9 ± 0.8	0.57
Subjective support	21.2 ± 1.1	23.0 ± 0.6	0.12	20.7 ± 1.3	22.1 ± 0.6	0.30
Availability	7.1 ± 0.3	8.1 ± 0.6	0.31	8.5 ± 0.9	7.7 ± 0.4	0.39
Depression (EPDS)	13.8 ± 1.1	9.9 ± 0.6	<0.001	8.6 ± 1.0	7.8 ± 0.5	0.41
Yes^a^	16 (50.0)	26 (27.7)	<0.05	4 (14.8)	11 (14.3)	1.00
No^a^	16 (50.0)	68 (72.3)		23 (85.2)	66 (85.7)	
Affect (PANAS)						
Positive affect	24.8 ± 0.9	30.3 ± 0.7	<0.0001	30.8 ± 1.1	30.3 ± 0.7	0.71
Negative affect	28.3 ± 1.3	24.3 ± 0.8	<0.05	23.9 ± 1.5	23.7 ± 0.9	0.91

*EPDS*, Edinburgh postnatal depression scale; *PANAS*, positive and negative affect scale; *PPS*, pregnancy pressure scale; *SCSQ*, simplified coping style questionnaire; *SSRS*, social support rating scale; *TPL*, threatened premature labor

^a^Data were presented as *n*(%)

Compared to their spouses, low resilient TPL women reported higher pressures from concerns of child support after delivery, less active coping, less positive affect and more negative affect. And high resilient women reported more social support (Additional file 1: Table S2). Additionally, TPL women in both groups had higher depression score and more proportion of depression compared to their partners (Additional file 1: Table S2).

Finally, there were positive correlations between resilience, active coping, positive affect and social support in TPL women. Negative correlations were also found in TPL women between depression, pressure, passive coping, negative affect and social support, and between depression, pressure and positive affect (Table 4). Notably, correlations of positive and negative affect with resilience were stronger in TPL women than in spouses (rho = 0.401 *vs* 0.243 and −0.296 *vs −*0.197, respectively), while correlation of negative affect with depression was stronger in spouses than in TPL women (rho = 0.636 *vs* 0.516) (Table 4). Furthermore, resilience of women had a modest negative correlation with negative affect of spouses (rho = −0.207), and the pressure of women had a modest positive correlation with the pressure and negative affect of spouses (rho = 0.243, 0.214, respectively) (Additional file 1: Table S3).

**Table 4:** Correlations between psychological factors of women with TPL and of spouses, respectively

	Resilience Pressure		Active coping	Passive coping	Social support	Depression	Positive affect	Negative affect
Women								
Resilience	1.000	-	-	-	-	-	-	-
Pressure	−0.347^b^	1.000	-	-	-	-	-	-
Active coping	0.337^b^	−0.049	1.000	-	-	-	-	-
Passive coping	−0.024	0.203^a^	0.165	1.000	-	-	-	-
Social support	0.327^b^	−0.215^a^	0.220^a^	−0.302^b^	1.000	-	-	-
Depression	−0.350^b^	0.434^b^	−0.089	0.075	−0.265^b^	1.000	-	-
Positive affect	0.401^b^	−0.215^a^	0.163	0.059	0.290^b^	−0.346^b^	1.000	-
Negative affect	−0.296^b^	0.446^b^	0.022	0.122	−0.231^b^	0.516^b^	−0.163	1,000
Spouses								
Resilience	1.000	-	-	-	-	-	-	-
Pressure	−0.375^b^	1.000	-	-	-	-	-	-
Active coping	0.306^b^	−0.005	1.000	-	-	-	-	-
Passive coping	−0.026	0.291^b^	0.184	1.000	-	-	-	-
Social support	0.148	0.006	0.185	−0.042	1.000	-	-	-
Depression	−0.316^b^	0.458^b^	−0.029	0.138	−0.092	1.000	-	-
Positive affect	0.243^a^	−0.076	0.281^b^	0.062	0.111	−0.154	1.000	-
Negative affect	−0.197^a^	0.492^b^	0.048	0.146	−0.082	0.636^b^	−0.002	1,000

*TPL*, threatened premature labor

^a^Correlation is significant at the 0.05 level (2-tailed)

^b^Correlation is significant at the 0.01 level (2-tailed)

## Discussion

The development of threatened premature labor (TPL) was a stressful and life threatening event in pregnancy for the families. It is not only a major adversity, but also a chronic stressor for that family due to the specific pathophysiological course of this complication in pregnancy, and family resilience plays a great role to buffer stress and provide support. However, the detailed information on family resilience of women with TPL was scarcely known.

In our study, social-demographic data were compared between women with different levels of resilience, their spouses and also within couples. We found the spouses of high resilient women were significantly older than their partners, as older spouses may have more life experience and coping skills to draw from when facing challenges such as TPL, thus could better support their partners in coping with chronic stress. Intriguingly, exclusively for high resilient TPL women, their spouses had higher proportions of income of 2000–2999 Yuan and lower proportions of 1000–1999 Yuan than themselves, suggesting the spouse income might be a protective factor for women resilience in lower income families. On the other hand, more spouses worked in enterprise or in the management position than high resilient TPL women, which may contribute to the high resiliency of women due to more economic support from their partners. Taken together, our data suggest that high resiliency of women could be attributable to the high socio-economic status of their spouses, but could also be explained by “economically comparative dominance” of their partners in the case of low socio-economic status. As these women may have a positive perception of one’s self, which has been found to act as a buffer for the detrimental effects of low socio-economic status [43]. And self-esteem may be thought of as an ego related resource which imparts a sense of mastery and competence when facing adversity, thus promotes family resilience [43].

The fact that higher proportion of placenta praevia was found in low resilient TPL women suggests this prenatal adverse outcome may be a risk factor of resilience for women with TPL. Indeed, preterm birth is a major cause of neonatal death and contributes significantly to newborn morbidity, including neonatal care complications, cerebral palsy, cognitive impairment, blindness, deafness, and respiratory illness [1–6]. These potential adverse outcomes, perceived by women with TPL, could lead to high levels of chronic psychosocial stress and negative affect accrued during the course of pregnancy, and gradually undermine their resiliency. On the other hand, shorter pediatric observation time was observed in high resilient TPL women at 6–8 weeks postpartum, suggesting resilience might be a protective factor of abnormal pregnancy. However, further study is needed to confirm this hypothesis.

The significant differences of component CD-RISC scores between low and high resilient TPL women testified the rationality of grouping in this study. Based on comparisons of resilient scores between TPL women and spouses, it also suggested that TPL has great impact on the resilience of partial pregnant women, with trivial impact on that of spouses. The differential reaction to preterm birth between mothers and fathers was also reported in recent studies by Provenzi and colleagues, who found that for couples with very preterm birth, mothers had moderate levels of adjustment to preterm birth and focused on awareness of their own maternal roles, while fathers had low to moderate levels of adjustment to preterm birth and limited assumption of paternal role [44]. Therefore, active engagement of both parents is advocated to promote family-centered care in families with preterm birth [45]. Although not explicitly tested, our results postulate the possibility that generalized anxiety disorder may exist in the low resilience TPL women, as the origin of their pressure had already exceeded their pregnancy complication, such as parenthood recognition, rear child properly, spouses mutual affection. Our data also suggest that low resilience of TPL women was primarily due to the lack of active coping rather than excessive passive coping. Indeed, adaptability and psychological resilience have been associated with active coping, which involves behavioral and/or psychological strategies to change qualities of the stressor, the stressor itself or how this is perceived [46]. Additionally, similar levels of pressure were found between low resilient TPL women and their partners, suggesting spouses of low resilient women bore equivalent pressure as their wives, possibly conducted from their partners, as maternal pressure was found to be positively correlated with paternal pressure in this study.

In our study the depression rates were 27.7% for high resilient women and 50% for low resilient women with TPL. Compared to the reported maternal perinatal depression rate ranging from 7 to 20% [11–15, 47–49], the prevalence of maternal antenatal depression in pregnancy complicated with TPL was exceedingly high, especially for low resilient women, and women with high resilience were also significantly impacted. Given the fact that 13 was the cutoff value of EPDS to screen for depression (the highest score in the literature to report probable depression), the probability of overestimation of the prevalence of depression is extremely low. Preliminary findings showed that depression affected 4.8 to 12% fathers in the antenatal period [19, 50], our results show that the situation in the case of TPL was even worse, as more than 14% spouses in this study had probable depression. The depression symptoms were also more severe in low resilient women than high resilient women with TPL. Altogether, the above results suggest a protective role of resilience in TPL women against depression, and a great effort to be made to early intervene into the depressive symptoms of families with TPL. Particularly, not only women with low resilience, but also their partners should be included.

Levels of social support were higher in high resilient women compared to their partners in this study. This is consistent with previous report that social support buffered women against the risk of antenatal depressive symptoms [51], as was also found here that high resilient women had lower EPDS scores.

In terms of correlations among psychometric factors examined in this study, our data suggest that generally the interactions were more complex in TPL women than in spouses. For example, we found exclusively in TPL women, social support was positively associated with resilience, active coping, and positive affect, and negatively associated with pressure, passive coping, negative affect, and depression. However, these associations were not found in spouses. This is consistent with previous report that high levels of social support were positively associated with active coping, resilience and lower levels of depression [52]. On the other hand, these results suggest dramatic differences in the interaction spectrums of psychological factors between TPL women and spouses, if extended, between different genders. Indeed, Cronenwett and Kunst-Wilson stated that men tended to have poorer social support networks compared to women, as men tend to rely primarily on their partners for support after getting married [53]. Of note, relatively simple and moderate associations between psychometric factors in spouses were found in this study. It may be due to the fact that males tend to hide emotions they experience in comparison to females, which also justified the under estimation of the rate of males’ mental health problems [54, 55]. Furthermore, for the first time, our results show that women’s resilience was negatively associated with spouses’ negative affect, and women’s pressure was positively correlated with spouses’ pressure and negative affect, suggesting psychological interactions exist within couples. Hence, interventions aiming at alleviating familial negative affect and pressure could be beneficial to promote resilience of both expectant mothers and fathers.

We are aware of a relatively small sample that limits the findings of this study due to time constraint and the great difficulties to collect data on both expectant mothers and fathers during an antenatal period of high risk pregnancy complicated by threatened premature labor. In considering of this limitation, we used Fisher’s exact test to measure the statistical power of significance whenever the frequencies were less than 5. Although this is a multi-centered, cross-sectional study, it should also be acknowledged that the data was collected from the inpatient unit of three major medical centers in only the core area in Chongqing. The sampling did not include subjects from primary or secondary antenatal clinics or hospitals in other districts or relatively rural areas in Southwest China. Caution should be exercised in generalizing the results to couples with high-risk pregnancies of other categories, or couples residing in other geographic areas of China. It should also be emphasized that EPDS is just a screening instrument, not a diagnostic tool, and the detected rates only indicate probable depression. Future studies should consider including a diagnostic interview and matched controls to confirm the clinical status and the prevalence rates of depression in the couples, as well as to provide a reliable comparison group.

## Conclusions

The current study found that more placenta praevia and longer pediatric observation were associated with low resilience for TPL women. Low resilient women also had higher pressure in pregnancy, less active coping, more depressive symptoms, higher rates of depression, less positive affect and more negative affect. Although TPL had trivial impact on most psychometric parameters of spouses, their pressure and depression should not be ignored. The present study also revealed different spectrum of interactions of psychometric factors for couples with TPL, with women’s resilience negatively correlated with spouses’ negative affect. These findings suggest that in addition to clinical treatment of high-risk pregnancies, particularly those complicated with placenta praevia, psychological screening and intervention for the detection of depression should be done as early as possible on TPL women and their partners as an integrity to better promote family resilience and their well-being, including the expectant child.

## Additional file

Additional file 1: Table S1. Socio-demographic data between women with TPL and spouses divided by level of resilience of women. **Table S2** Psychometric data between women with TPL and spouses divided by level of resilience of women. **Table S3** Correlations between psychological factors of women with TPL and spouses. (DOCX 20 kb)

## Abbreviations

CD-RISC: Connor-Davidson resilience scale
EPDS: Edinburgh postnatal depression scale
GDM: Gestational diabetes mellitus
ICP: Intrahepatic cholestasis of pregnancy
IUI: Intrauterine insemination
IVF: In-vitro fertilization
PANAS: Positive and negative affect scale
PPS: Pregnancy pressure scale
PROM: Premature rupture of membranes
SCSQ: Simplified coping style questionnaire
SSRS: Social support rating scale
TMMU: Third military medical university
TPL: Threatened premature labor
TSS: Technical secondary school
