# Supplementary material for: The impact of resilience on psychological outcomes in women with threatened premature labor and spouses: a cross-sectional study in Southwest China
Source: Health Qual Life Outcomes. 2017 Jan 31;15:26. doi: 10.1186/s12955-017-0603-2 (PMC5282797; doi:10.1186/s12955-017-0603-2)
Supplement: Additional file 1: Table S1. — Socio-demographic data between women with TPL and spouses divided by level of resilience of women. Table S2 Psychometric data between women with TPL and spouses divided by level of resilience of women. Table S3 Correlations between psychological factors of women with TPL and spouses. (DOCX 20 kb) [file 12955_2017_603_MOESM1_ESM.docx]

**Table S1 Socio-demographic data between women with TPL and spouses divided by level of resilience of women**

| Low resilient Spouses of low High resilient Spouses of high  women resilient women women resilient women  (n = 32) (n = 27) *p* (n = 94) (n = 77) *p*  n (%) n (%) n (%) n (%) | | | | | | |
| --- | --- | --- | --- | --- | --- | --- |
| Age (years)  Height (cm)  Weight (kg)  Smoker  Yes  No  Drinker  Yes  No  Residence  Urban  Rural  Education  Middle school  High school/TSS  Junior college  University  Monthly income  < 1000  1000-1999  2000-2999  3000-4999  5000-9999  Others  Occupation categories  Government/Military Enterprise/Management  Office  Education/Science  Healthcare  Industry/Service  Private business  Others | 30.7 ± 0.9  159.1 ± 0.8  63.8 ± 1.6  0 (0.0)  32 (100)  0 (0.0)  32 (100)  21 (65.6)  11 (34.4)  5 (15.6)  6 (18.7)  15 (46.9)  6 (18.8)  3 (9.4)  7 (21.9)  8 (25.0)  10 (31.2)  0 (0.0)  4 (12.5)  2 (6.2)  2 (6.2)  6 (18.8)  4 (12.5)  0 (0.0)  3 (9.4)  2 (6.2)  13 (40.7) | 31.4 ± 0.9  167.9 ± 1.3  67.7 ± 2.1  14 (51.9)  13 (48.1)  12 (44.4)  15 (55.6)  19 (70.4)  8 (29.6)  4 (14.8)  5 (18.6)  9 (33.3)  9 (33.3)  0 (0.0)  3 (11.1)  7 (25.9)  9 (33.3)  5 (18.6)  3 (11.1)  1 (3.7)  6 (22.2)  8 (29.6)  3 (11.1)  0 (0.0)  3 (11.1)  1 (3.7)  5 (18.6) | 0.55  < 0.0001  0.13    < 0.0001  < 0.0001    0.78  1.00  0.98  0.29  0.20  0.24  0.32  0.94  0.86  < 0.05  1.00  1.00  0.13  0.33  1.00  1.00  1.00  1.00  0.07 | 29.4 ± 0.4  158.2 ± 1.2  65.2 ± 0.9  6 (6.4)  88 (93.6)  5 (5.3)  89 (94.7)  67 (71.3)  27 (28.7)  16 (17.0)  25 (26.6)  35 (37.2)  18 (19.2)  8 (8.5)  18 (19.1)  18 (19.1)  27 (28.7)  8 (8.5)  15 (16.1)  5 (5.3)  4 (4.3)  24 (25.5)  8 (8.5)  1 (1.1)  18 (19.2)  9 (9.6)  25 (26.6) | 31.2 ± 0.8  167.4 ± 1.6  69.3 ± 1.8  42 (54.5)  35 (45.5)  38 (49.4)  39 (50.6)  50 (64.9)  27 (35.1)  12 (15.5)  26 (33.8)  19 (24.7)  20 (26.0)  2 (2.6)  5 (6.5)  25 (32.4)  20 (26.0)  15 (19.5)  10 (13.0)  2 (2.6)  12 (15.6)  15 (19.5)  6 (7.8)  3 (3.9)  11 (14.3)  12 (15.5)  16 (20.8) | < 0.05  < 0.0001  < 0.05    < 0.0001    < 0.0001  0.37  0.80  0.31  0.08  0.29  0.19  < 0.05  < 0.05  0.69  < 0.05  0.58  0.46  < 0.05  0.35  0.86  0.33  0.40  0.23  0.38 |

TPL, threatened premature labor; TSS, technical secondary school.

**Table S2 Psychometric data between women with TPL and spouses divided by level of resilience of women**

| Low resilient Spouses of low High resilient Spouses of high  women resilient women women resilient women  (n = 32) (n = 27) *p* (n = 94) (n = 77) *p*  n (%) n (%) n (%) n (%) | | | | | | |
| --- | --- | --- | --- | --- | --- | --- |
| Pregnancy pressure (PPS)  Parenthood recognition  Health/safety of mother /fetus  Body shape/activity change  Other factors  Rear child properly  Spouses mutual affection  Child support  Coping style (SCSQ)  Active coping  Passive coping  Social support (SSRS)  Objective support  Subjective support  Availability  Depression (EPDS)  Yes^*^  No^*^  Affect (PANAS)  Positive affect  Negative affect | 65.4 ± 2.5  26.6 ± 1.1  24.8 ± 1.0  9.6 ± 0.7  2.6 ± 0.2  1.7 ± 0.1  2.7 ± 0.2  19.1 ± 0.9  11.1 ± 0.8  38.4 ± 1.2  9.9 ± 0.9  21.2 ± 1.1  7.1 ± 0.3  13.8 ± 1.1  16 (50.0) 16(50.0)  24.8 ± 0.9  28.3 ± 1.3 | 60.3 ± 3.0  23.2 ± 1.3  22.0 ± 1.4  9.1 ± 0.9  2.2 ± 0.2  1.4 ± 0.1  2.1 ± 0.2  22.3 ± 1.1  12.4 ± 1.1  40.2 ± 1.6  11.8 ± 1.2  20.7 ± 1.3  8.5 ± 0.9  8.6 ± 1.0  4 (14.8)  23 (85.2)  30.8 ± 1.1  23.9 ± 1.5 | 0.19  0.05  0.10  0.62  0.17  0.12  < 0.05  < 0.05  0.33  0.38  0.21  0.77  0.12  < 0.001  < 0.01  < 0.0001  < 0.05 | 56.1 ± 1.5  22.1 ± 0.7  21.0 ± 0.7  8.3 ± 0.4  2.0 ± 0.1  1.4 ± 0.1  1.9 ± 0.1  22.5 ± 0.6  11.3 ± 0.5  41.3 ± 0.8  10.7 ± 0.4  23.0 ± 0.6  8.1 ± 0.6  9.9 ± 0.6  26 (27.7)  68 (72.3)  30.3 ± 0.7  24.3 ± 0.8 | 54.3 ± 1.6  21.1 ± 0.7  20.1 ± 0.8  8.5 ± 0.7    2.3 ± 0.2  1.4 ± 0.1  1.9 ± 0.1  21.8 ± 0.9  11.9 ± 0.7  38.5 ± 1.0  10.9 ± 0.8  22.1 ± 0.6  7.7 ± 0.4  7.8 ± 0.5  11 (14.3)  66 (85.7)  30.3 ± 0.7  23.7 ± 0.9 | 0.40  0.37  0.39  0.83  0.27  0.87  0.88  0.48  0.55  < 0.05  0.83  0.27  0.57  < 0.01  < 0.05  0.94  0.62 |

EPDS, Edinburgh postnatal depression scale; PANAS, positive and negative affect scale; PPS, pregnancy pressure scale; SCSQ, simplified coping style questionnaire; SSRS, social support rating scale; TPL, threatened premature labor. ^*^ Data were presented as n(%).

**Table S3 Correlations between psychological factors of women with TPL and spouses**

|  | | Women  Resilience Pressure Active Passive Social Depression Positive Negative  coping coping support affect affect | | | | | | | |
| --- | --- | --- | --- | --- | --- | --- | --- | --- | --- |
| Spouses  Resilience  Pressure  Active coping  Passive coping  Social support  Depression  Positive affect  Negative affect | 0.046  -0.175  0.006  -0.149  0.054  -0.132  -0.019  -0.207^*^ | | -0.063  0.243^*^  -0.012  -0.038  -0.058  0.062  -0.015  0.214^*^ | 0.050  -0.189  0.129  -0.038  0.193^*^  -0.080  -0.122  -0.080 | 0.068  0.007  -0.075  -0.174  0.010  0.084  0.161  0.145 | -0.090  -0.029  -0.054  0.073  0.063  -0.067  -0.032  -0.165 | -0.164  0.165  -0.067  -0.002  -0.040  -0.006  -0.022  0.003 | 0.047  -0.123  -0.144  0.036  0.088  -0.042  -0.069  0.119 | -0.097  0.096  -0.156  -0.022  -0.026  0.171  0.020  0.124 |

TPL, threatened premature labor; ^*^Correlation is significant at the 0.05 level (2-tailed).
